# Evaluation of C-C Chemokine Ligand 5 (CCL5) Chemokine, Interleukin 5 (IL-5) Cytokine, and Eosinophil Counts as Potential Biomarkers in Saudi Patients with Chronic Asthma During Sandstorms

**DOI:** 10.7759/cureus.7809

**Published:** 2020-04-24

**Authors:** Wael H Alturaiki

**Affiliations:** 1 Department of Medical Laboratory Sciences, College of Applied Medical Sciences, Majmaah University, Majmaah, SAU

**Keywords:** asthma, sandstorms, total ige, ccl5, il-5, eosinophil

## Abstract

Background and objectives

Asthma is a common chronic inflammatory disorder of the lung that can be exacerbated by environmental triggers during sandstorms. This study aimed to evaluate the usefulness of C-C chemokine ligand 5 (CCL5) chemokine and interleukin 5 (IL-5) cytokine and determine the total eosinophil count in blood and sputum for use as biomarkers in Saudi patients with chronic asthma who visited emergency departments during sandstorms.

Methods

The study included 42 Saudi patients with chronic asthma and 20 healthy controls. Plasma levels of CCL5, IL-5, and total immunoglobulin E (IgE) were measured using a specific enzyme-linked immunosorbent assay (ELISA). Total eosinophils in peripheral blood were counted using a hematology analyzer (CELL-DYN Ruby System; Abbott Diagnostics, Chicago, Illinois); in sputum, eosinophils stained with Giemsa were examined under a microscope, counted, and expressed as a percentage of the total cells.

Results

Total IgE levels were significantly higher in patients with asthma (mean 433 IU/ml, P = 0.0001) as compared to normal controls (139 IU/ml). There was no significant difference in the levels of CCL5 in patients with asthma (625 pg/ml) as compared to normal controls (663 pg/ml, P = 0. 57). No correlation was found between total IgE and CCL5 levels. IL-5 was not detected in patients with asthma or in controls. Moreover, the total counts of eosinophils in the blood did not increase in patients with asthma as compared to controls while eosinophils in sputum samples were increased in the former (mean =3.128%).

Conclusion

Plasma levels of CCL5 and IL-5 or eosinophil counts in the peripheral blood may not be useful diagnostic biomarkers to evaluate airway inflammation and monitor asthma severity. Conversely, the sputum eosinophil count may represent a useful diagnostic marker for assessing the magnitude of asthma exacerbation during sandstorms.

## Introduction

Asthma is a common chronic inflammatory disorder of the lung characterized by bronchial hyperresponsiveness with clinical symptoms, including wheezing, coughing, mucus production, migration of inflammatory immune cells into airways, and shortness of breath [1]. According to the World Health Organization (WHO), approximately 235 million individuals worldwide have asthma [2]. Asthma is considered one of the most common chronic diseases in Saudi Arabia, affecting more than 2 million people over the past three decades, possibly as a result of changes in lifestyle, dietary habits, and exposure to environmental factors such as dust, indoor allergens, tobacco, and sandstorms [3].

Major sandstorms are common in the Middle East [4]. During these sandstorms, patients with asthma are at a very high risk of disease exacerbation. Acute asthma exacerbations are considered one of the most common reasons for emergency room visits, and such exacerbation can be initiated by exposure to dust or during sandstorms [5-6]. These sandstorms carry some of the most important allergenic fungal spores that may initiate an allergic reaction in patients with asthma, as reported in our previous study [7].

Atopic asthma is classified as a type-1 hypersensitivity, where immunoglobulin E (IgE) has a crucial function in the allergic reaction [8]. Eosinophils play a key role in asthma, and these cells have been shown to contribute to disease exacerbation [9]. Accordingly, there is a need for better markers of disease exacerbation in patients exposed to sandstorms.

Regulated upon activation, normal T-cell expressed and secreted (RANTES), a chemokine also known as C-C chemokine ligand 5 (CCL5), is a member of the CC chemokine family with a molecular weight of 7.5 kDa that plays an important role in the inflammatory process [10]. CCL5 is a potent leucocyte chemoattractant that upon interaction with its receptors, CCR1, CCR3, and CCR5, induces the activation and trafficking of a wide range of immune cells, including T-cells, monocytes, basophils, eosinophils, natural killer (NK) cells, and dendritic cells (DCs), to the site of inflammation [10]. Increased levels of CCL5 have been reported in patients with asthma [10-11]. Furthermore, targeting CCL5 with antibodies in a murine model of allergic airway disease was shown to inhibit airway inflammation [12].

Interleukin 5 (IL-5) is a Th2 cytokine with a molecular weight of approximately 52 kDa that has also been shown to play an important role in the pathogenicity of asthma. IL-5 interacts with its receptor (IL-5Ra) expressed on eosinophils, maintaining airway inflammation and resulting in worsened asthma symptoms. Targeting IL-5 or its receptor has been shown to be a promising therapeutic approach for severe asthma [13]. Based on the above findings, CCL5, IL-5, and eosinophils clearly play a key role in the development of asthma.

In this study, the plasma levels of the chemokine CCL5 and cytokine IL-5 and total IgE and eosinophil counts in the peripheral blood and sputum were investigated in patients with asthma and normal controls during sandstorms to assess their possible use as biomarkers to evaluate disease exacerbation in patients exposed to sandstorms.

## Materials and methods

Subjects

Forty-two people with allergic asthma (mean age 37.06 years) and 20 normal controls (mean age 31.08 years) were enrolled in this study. The characteristics of the subjects with asthma are shown in Table 1. Asthma was diagnosed according to the Saudi Initiative for Asthma guidelines [14]. The patients were undergoing treatment, including inhaled corticosteroids (ICS) with or without a long-acting beta-agonist (LABA). Samples were collected from patients with chronic symptoms of asthma who visited hospital emergency departments during sandstorms between April 2016 and May 2017. All patients with asthma were examined, and their clinical status was determined by specialty physicians at Al Zulfi General Hospital, Kingdom of Saudi Arabia. All patients with asthma included in this study were non-smokers or had no respiratory tract infection prior to and during the study. The normal controls were recruited and selected from healthy blood donors from the blood bank at Al Zulfi General Hospital and were defined as those who had no history of any allergic diseases and no signs of wheezing, did not smoke or have any other chronic diseases, respiratory infection, or recent vaccinations, and were not receiving treatment. The study was approved by the Majmaah University Ethical Committee (approval no. MUREC-April.01/COM-206), and written informed consent was obtained from all subjects.

**Table 1:** Characteristics of subjects ICS: Inhaled corticosteroid; LABA: Long-acting beta-agonist

Categories	Control subjects	Subjects with asthma
Number of subjects	20	42
Sex (male/female)	11/9	23/19
Mean age	31.08	37.06
Duration of asthma (years)	-	1 to >30
ICS usage LABA usage		36 6
Clinical presentation at the time of sampling		
Shortness of breath	No	Increased
Cough	A symptomatic coughing	Continuing
Wheezing	Normal	Frequently loud
Chest tightness	No	Present
Mucus production	Not present	Increased
Respiratory tract infection (viral, bacterial, and fungal)	Not present	Not present
Speaking	Normal	Difficult
Mean of heart rate per minute	96.06	109.07
Mean of O_2_ saturation %	95.08	90.5

Methods

Blood Collection and Isolation

Peripheral blood was collected from healthy donors and patients. Following collection, the blood was transferred immediately to tubes containing the anticoagulant ethylenediaminetetraacetic acid (EDTA). The blood was centrifuged at 1800 rpm for 5 min at room temperature. After centrifugation, the plasma aspirated and divided into aliquots in small plastic tubes.

Eosinophil Counts in Peripheral Blood and Sputum

After collection in EDTA tubes, a hematology analyzer (CELL-DYN Ruby System; Abbott Diagnostics, Chicago, Illinois) was used to determine the complete blood counts (CBCs) and differential counts of white blood cells (WBCs), including eosinophils. Blood eosinophilia was identified at a cutoff of 5% and/or >300 cells/mm^3^. The sputum samples were processed as follows. Briefly, all study subjects were requested to cough sputum into plastic containers. The sputum samples were examined macroscopically to ensure that they were free of salivary contaminants. Satisfactory sputum samples were treated with dithiothreitol (1% Dithiothreitol (DTT)) in phosphate-buffered saline for 20 min to homogenize and disrupt the disulfide bonds in the mucous and disperse the cells. The suspension was centrifuged, and the cell pellet was resuspended, transferred, and distributed thinly and evenly over a microscope slide. Giemsa staining was performed, and the number of eosinophils was assessed under a microscope. The eosinophil count is expressed as a percentage (%). A sputum eosinophil count ≥2% of total leukocytes in a sputum sample was considered abnormal based on the Saudi Initiative for Asthma guidelines (2019) [14].

Cytokine Measurement by ELISA

The plasma obtained from the healthy controls and patients was used for measuring protein concentrations using specific ELISAs. Total IgE levels were measured using Quantikine ELISAs (Abcam, Cambridge, UK, cat# ab108650). Human RANTES or CCL5 (R&D, UK, cat# DRN00B) and IL-5 (R&D, UK, cat# D5000B) levels were also measured using Quantikine ELISAs. The assays were conducted as described in the manufacturer’s instructions for each kit. The optical density (OD) of each well at 450 nm was determined using a microplate reader (BioTek ELx800 Absorbance Microplate Reader; BioTek Company, Winooski, Vermont). KC Junior software (BioTek) was used to determine the final protein concentrations.

Statistics

Data are expressed as mean ± standard deviation (SD), and an independent nonparametric sample test (Mann-Whitney U test) was employed to evaluate statistical significance using GraphPad Prism 6 software (GraphPad Software, San Diego, California). Pearson’s test was applied to evaluate correlations. P-values <0.05 indicate significance.

## Results

Total IgE was significantly increased in patients with asthma as compared to normal controls

**Figure 1:**
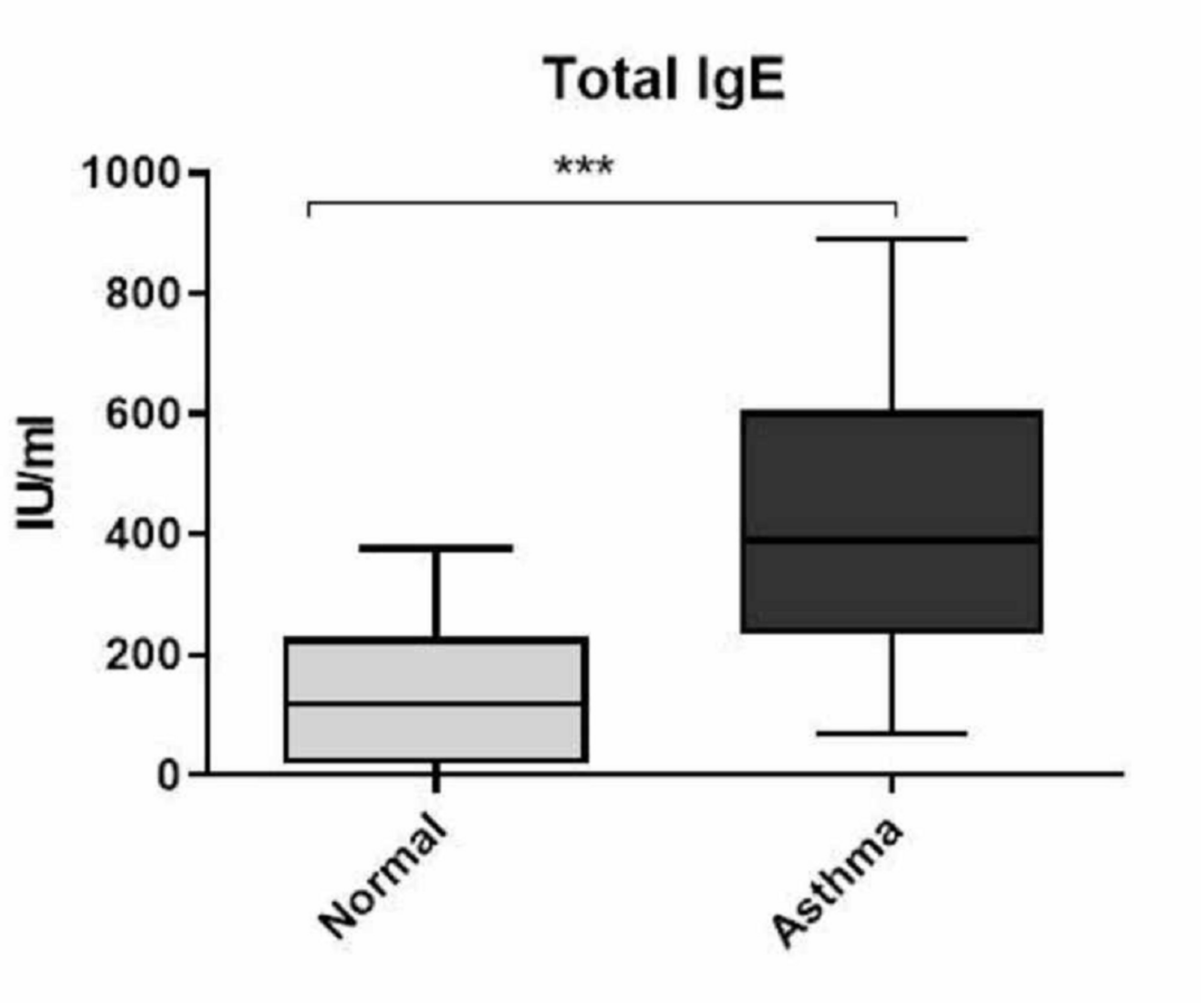
Total IgE levels in the plasma Total IgE levels were measured using a specific ELISA. Total IgE levels were significantly elevated in patients with asthma compared to normal controls. Data are expressed as the mean ± standard deviation (SD). ***P<0.0001. IgE: immunoglobulin E; ELISA: enzyme-linked immunosorbent assay

Atopic asthma is an allergic disease that is characterized by increased levels of IgE. To ensure that the samples received from the hospital were from patients with allergic asthma, plasma IgE levels were measured using an ELISA. Total IgE levels were significantly elevated in all patients with asthma (mean 433 IU/ml) (P=0.0001) as compared to normal controls (139 IU/ml) (Figure 1).

CCL5 chemokine protein levels were not significantly increased in patients with asthma as compared to normal controls

**Figure 2:**
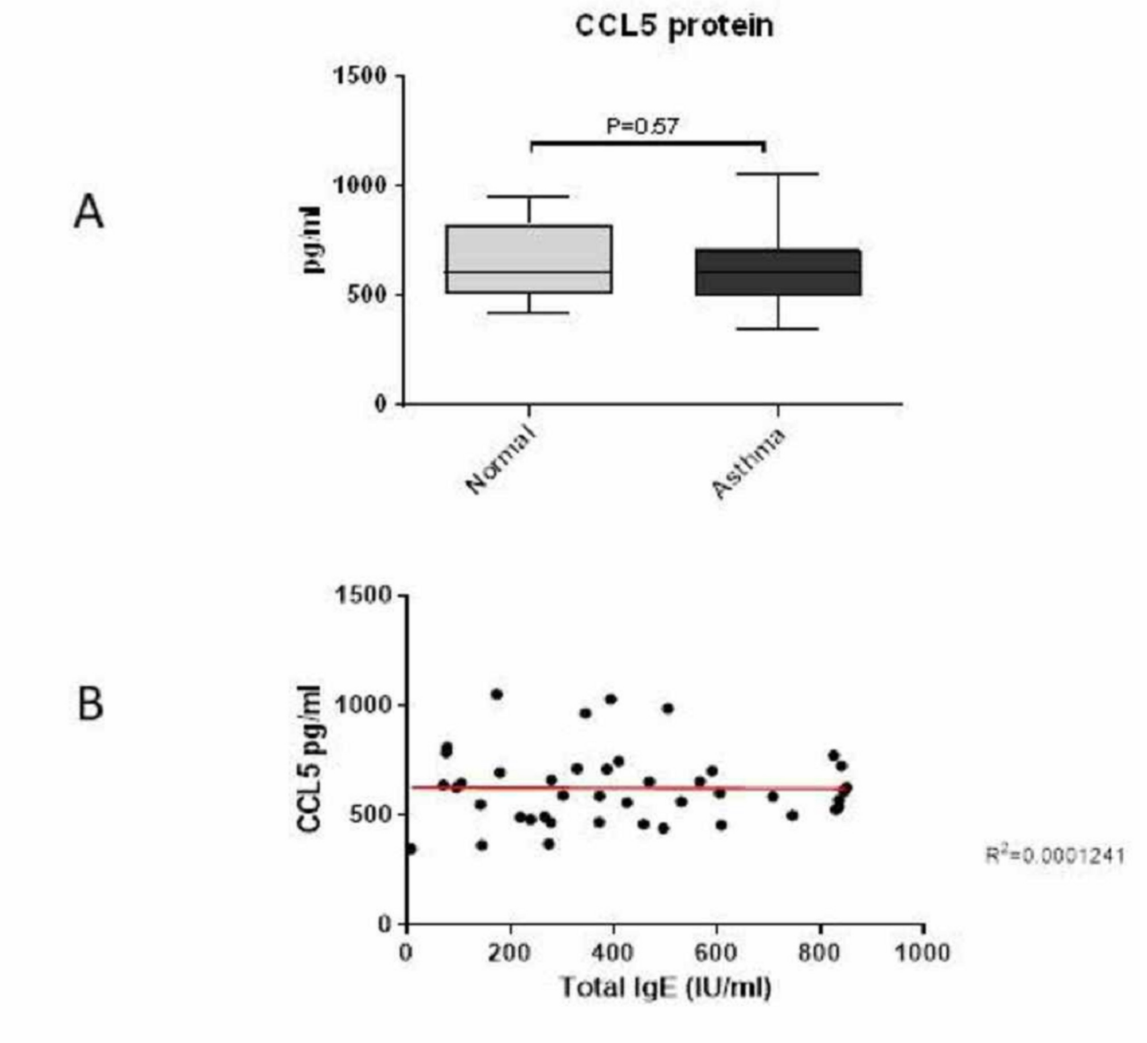
Plasma levels of CCL5 chemokine and correlation with total IgE CCL5 chemokine levels were measured using a specific ELISA. No significant increase in CCL5 protein levels was observed in patients with asthma as compared to normal controls (A). Data are expressed as mean ± standard deviation (SD). *P<0.05. No correlation was found between CCL5 and total IgE in the plasma of patients with asthma based on Pearson’s test (B). ELISA: enzyme-linked immunosorbent assay; IgE: immunoglobulin E

No significant difference was observed in the levels of CCL5 chemokine between the patients (625 pg/ml) and normal controls (663 pg/ml) (P=0.57) (Figure 2A). Furthermore, no correlation was found between CCL5 and total IgE in the plasma of patients with asthma (Figure 2B).

IL-5 cytokine was not detected, and total eosinophil counts were not increased in blood but in the sputum

**Figure 3:**
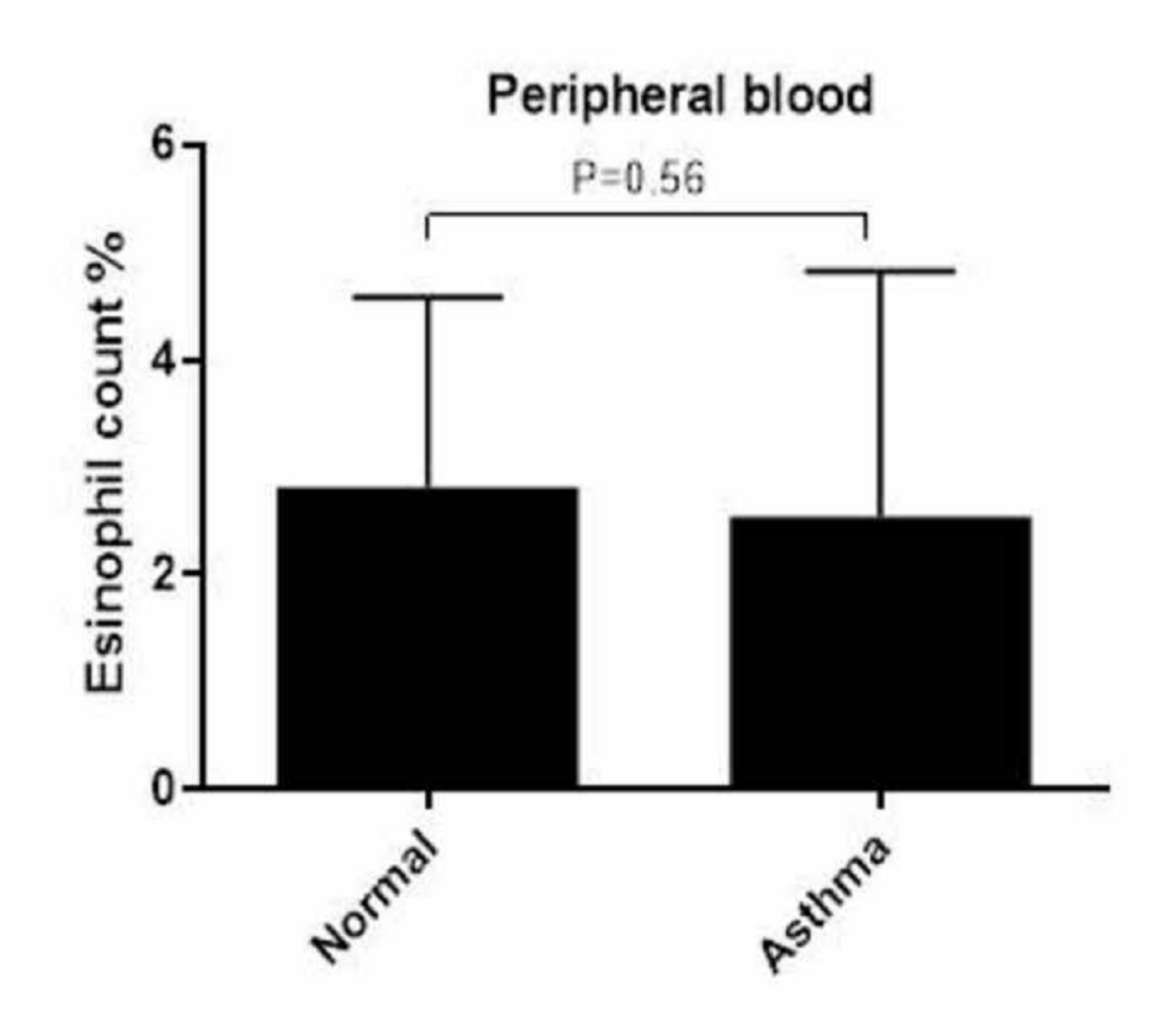
Eosinophil counts in the peripheral blood No significant increase in eosinophils in the peripheral blood was observed in patients with asthma or normal controls. Data are expressed as mean ± standard deviation (SD).

IL-5 was not detected in the plasma of normal controls or patients with asthma. The eosinophil count in the peripheral blood of the patients was within the normal range (mean 2.55%) and was not significantly different when compared to that of the normal controls (mean 2.81%) (Figure 3). However, the eosinophil count in the sputum of the patients with asthma was higher (mean =3.128%) than 2% because this percentage was considered abnormal based on the Saudi Initiative for Asthma guidelines [14].

## Discussion

This study aimed to evaluate plasma CCL5 (chemokine) and IL-5 (cytokine) as well as eosinophils in peripheral blood and sputum and their possible role in patients with chronic asthma who visited emergency rooms during sandstorms.

Unsurprisingly, a significant increase in total IgE was observed in patients with asthma (mean 433 IU/ml) as compared to normal controls (139 IU/ml). The normal levels of total IgE values show a wide range of distribution. Total IgE levels (≥100 IU/mL) have been used to determine atopic status [10]. However, in the Saudi population, it has been reported that 10% of individuals have values (≥200 IU/ml) [15]. This is also in agreement with the current finding of the total IgE levels in the normal controls. This variation between the Saudi and Western populations may be explained as differences related to genetic, racial, and environmental factors [15].

In addition, there was no significant increase in CCL5 levels in the plasma of patients with asthma (mean 641 pg/ml) relative to that in the healthy controls (588 pg/ml). Furthermore, no correlation was found between CCL5 and total IgE.

This finding is similar to that of Erten et al., who found no difference in CCL5 levels in patients with asthma relative to normal controls [16]. This finding may result from the inhaled corticosteroids used by these patients, which have been reported to suppress CCL5 via the inhibition of NF-κB-dependent transcription [17]. Other studies have also reported that CCL5 levels are significantly increased in the serum or plasma of patients with asthma who did not receive treatment prior to the study as compared to normal controls, with positive correlations with the severity score, total eosinophil count, and total serum IgE [10,18]. However, targeting the chemokine CCL5 with antibodies in a murine model of allergic airway disease resulted in the inhibition of airway inflammation, suggesting the importance of CCL5 in determining the severity of asthma [12].

Taken together, these results suggest that CCL5 may not be a useful marker of asthma severity during sandstorms, especially if the patients used ICS treatments during the storms. Furthermore, anti-CCL5 treatment would not be a useful therapeutic approach for this patient group.

IL-5 plays a key role in eosinophil growth, differentiation, activation and effector function, and survival [19]. It has been reported that IL-5 levels in serum can be used as a biomarker for the blood eosinophilia asthma phenotype and that targeting IL-5 or its receptor alpha subunit (IL-5Ra) reduces eosinophil numbers and disease severity [20-21].

In this study, IL-5 was not detected in the plasma of patients with asthma or normal controls. The lower detection limit for IL-5 in the ELISA is 25 pg/ml, and most studies have shown a range between 5 pg/ml and 20 pg/ml, which is below the detection limit in the current study; therefore, the cytokine would not be detected. Moreover, treating asthma patients with ICS has been shown to suppress the transcription of the gene encoding IL-5 [22]. This finding suggests that IL-5 may not be a useful biomarker to monitor asthma progression if patients use ICS during sandstorms.

It has been proposed that blood eosinophil counts reflect the severity of asthma [23]. In addition, Durham et al. demonstrated the relationship between the number and action of blood eosinophils with the asthmatic response after allergen challenge, suggesting that elevated blood eosinophil counts correlate with airway inflammation in asthma [24]. Thus, blood eosinophil counts have been proposed as an indirect marker of airway inflammation in asthma [25]. Conversely, it has been shown that the total count of eosinophils in the blood cannot be used as a biomarker due to the variability of these cells in patients with asthma, which could not be explained by seasonality or diurnal variation [26-27]. This latter study is consistent with the findings of the current study, whereby no increase in eosinophil counts in the blood in patients with asthma relative to normal controls was observed. Again, these results can be attributed to the use of corticosteroids, which have been shown to inhibit eosinophils [28].

Interestingly, the total number of eosinophils in the sputum of patients with asthma patients was increased as compared to the normal range. Despite the importance of ICS in reducing sputum eosinophils and asthma exacerbations, the variability in response among patients with asthma must still be considered, as not all of these patients respond equally to ICS [29-30].

Taken together, the observations suggest that the eosinophil count in sputum, but not in blood, maybe a marker for predicting the severity of disease during asthma exacerbations and whether patients require higher doses of ICS to manage their asthma severity.

The limitations of the current study are that asthmatic patients with high plasma levels of total IgE were not examined for further evidence of allergic bronchopulmonary aspergillosis or parasitic infection. In addition, the analysis of CCL5 and IL-5 in the sputum was outside the scope of this work, and thus, consequent studies should investigate the probability that sputum cytokine expression is a good indicator of asthma activity.

## Conclusions

In conclusion, the plasma levels of CCL5 and IL-5, as well as the eosinophil count in peripheral blood, may not be useful diagnostic biomarkers to evaluate airway inflammation and monitor asthma severity during sandstorms, especially if the patients have used ICS. However, the eosinophil count in the sputum may represent a useful diagnostic marker for asthma exacerbation.
